# Perceived physical education teacher autonomy support and intention to be physically active among Chinese senior high school students: cross-sectional serial indirect associations involving physical activity enjoyment and exercise identity

**DOI:** 10.3389/fpsyg.2026.1935171

**Published:** 2026-09-24

**Authors:** Tiantian Yuan, Hui Zhu, Yang Jiao, Lintong Gan

**Affiliations:** 1 Luoyang Vocational College of Culture and Tourism, Luoyang, Henan, China; 2 Myongji University, Yongin, Gyeonggi-do, Republic of Korea

**Keywords:** Chinese senior high school students, exercise identity, intention to be physically active, perceived physical education teacher autonomy support, physical activity enjoyment, physical education, serial indirect associations

## Abstract

**Background:**

Perceived physical education teacher autonomy support may be associated with students’ intentions to be physically active, but the distinct and joint roles of physical activity enjoyment and exercise identity remain unclear. This study examined specific and serial indirect associations involving these constructs and whether conclusions depended on mediator ordering.

**Methods:**

A cross-sectional survey included 1,824 students from four general senior high schools in Luoyang, Henan Province, China. Participants completed 29 self-report items assessing the four constructs. Maximum-likelihood latent-variable structural equation modeling was the primary analysis. The hypothesized ordering, with physical activity enjoyment before exercise identity, and the reversed ordering were evaluated using 5,000 percentile bootstrap resamples. A composite-score regression-based serial indirect-effects analysis adjusted for gender, age, and grade served as a robustness check.

**Results:**

The hypothesized-order model fit well, with a comparative fit index of 0.996, a root mean square error of approximation of 0.013, and a standardized root mean square residual of 0.016. Perceived physical education teacher autonomy support showed positive total and direct associations with intention to be physically active, with standardized estimates of 0.488 and 0.295, respectively. The specific indirect associations through physical activity enjoyment and exercise identity were both nonzero, with a standardized estimate of 0.084 and a 95% confidence interval of [0.058, 0.111] for each. The serial indirect association through physical activity enjoyment followed by exercise identity was nonzero, with a standardized estimate of 0.025 and a 95% confidence interval of [0.017, 0.035]. The reversed-order model had identical fit and produced a nonzero serial indirect association, with a standardized estimate of 0.040 and a 95% confidence interval of [0.027, 0.054]. The hypothesized-order model explained 32.6% of the variance in intention to be physically active, and the robustness analysis was directionally consistent.

**Conclusion:**

Perceived physical education teacher autonomy support, physical activity enjoyment, and exercise identity were statistically associated with intention to be physically active. Because both mediator orderings were compatible with the cross-sectional data, the findings do not establish a unique temporal or causal sequence; the outcome reflects intention rather than actual physical activity behavior.

## 1 Introduction

The senior high school years represent an important developmental period during which students’ physical activity intentions and health-related behavior patterns continue to evolve. Intention to be physically active (IPA) reflects students’ intention to continue participating in physical activity and remain physically active in the future. It is an important psychological construct for understanding adolescents’ subsequent physical activity participation. A systematic review of physical education (PE) pedagogical models reported mixed findings for physical activity but generally positive findings for IPA, highlighting the potential relevance of school PE to both students’ current physical activity and their future physical activity intentions (Saiz-González et al., 2024). In China, research on a healthy PE curriculum model has highlighted the practical value of using school PE to support students’ extracurricular sport participation and related participation intentions (Hao and Yang, 2022). However, how perceived physical education teacher autonomy support (PPETAS) is associated with IPA among Chinese senior high school students, and which psychological pathways may be involved in this association, remain insufficiently understood.

In PE settings, PPETAS reflects the extent to which students perceive that their PE teachers provide opportunities for choice, acknowledge their perspectives, offer encouragement, and facilitate active participation. Theoretically, higher levels of PPETAS may be associated with stronger IPA. However, examining only the direct association between these constructs may not fully capture the psychological processes potentially involved. Physical activity enjoyment (PAE) reflects the pleasure, interest, and positive experiences that students derive from physical activity. Michael et al. (2016) found that participation in a greater variety of physical activities was associated with higher enjoyment and greater physical activity participation among high school students. Whereas PAE captures an affective experience associated with participation, exercise identity (EI) reflects the extent to which students incorporate exercise into their self-concept. PAE and EI may represent conceptually distinct affective and identity-based correlates of the PPETAS–IPA association. The unresolved question is whether each shows a specific indirect association when considered simultaneously and whether a theory-specified PAE-to-EI ordering yields a distinct serial indirect association in addition to the two specific pathways. Accordingly, this study aimed to (1) examine the association between PPETAS and IPA and (2) assess the specific indirect associations through PAE and EI, together with the hypothesized serial indirect association with PAE specified before EI. Given the cross-sectional design, a reversed-order model was also evaluated as an alternative statistical specification. In doing so, the study sought to clarify the distinct and joint statistical roles of affective experience and exercise-related self-definition, rather than establish a temporal or causal mechanism, in Chinese senior high school PE contexts.

## 2 Literature review and research hypotheses

### 2.1 Perceived PE teacher autonomy support and intention to be physically active

PPETAS refers to the extent to which students perceive that their PE teachers support their autonomous engagement in physical activity by offering meaningful choices, acknowledging students’ perspectives, providing encouragement and informational feedback, and explaining the rationale and value of physical activity (Reeve and Jang, 2006; Hagger et al., 2007). In PE settings, autonomy support extends beyond general teacher care by focusing specifically on whether students perceive that their choices, interests, and feelings are acknowledged and respected. IPA reflects students’ intention to continue participating in physical activity and to remain physically active outside PE classes or after graduation (Hein et al., 2004). The trans-contextual model of motivation provides relevant theoretical background for expecting an association between PPETAS and IPA. It proposes that perceived autonomy support in PE is linked to out-of-school physical activity intentions through autonomous motivation across PE and leisure-time contexts and belief-based determinants of intention (Hagger et al., 2003; Hagger and Chatzisarantis, 2016). Because these intervening motivational constructs were not assessed, the present study does not constitute a test of the full trans-contextual model; rather, the model is used as a broader conceptual framework for the expected PPETAS–IPA association.

Empirical studies have also linked perceived autonomy support in PE to conceptually related measures of intention to engage in physical activity. A cross-cultural evaluation found broad, although not uniform, support for the core motivational sequence of the trans-contextual model across high school samples from Britain, Greece, Poland, and Singapore (Hagger et al., 2005). In a sample of secondary school students in Singapore, perceived autonomy support in PE was associated with more self-determined behavioral regulations, which were in turn related to intentions to be physically active outside school (Lim and Wang, 2009). In a sample of university students enrolled in PE courses, perceptions of an autonomy-supportive teacher style were positively associated with intentions to continue participating in physical activity beyond PE classes, with need satisfaction and autonomous motivation forming part of the proposed motivational process (Behzadnia et al., 2018). These studies provide conceptually relevant empirical support for an association between perceived autonomy support in PE and physical activity intentions; however, their populations, motivational constructs, and intention measures differ from those examined in the present study. Accordingly, the following hypothesis was proposed:

*H1*: Perceived PE teacher autonomy support will be positively associated with intention to be physically active.

### 2.2 Hypothesized indirect association through physical activity enjoyment

PAE refers to the pleasure, interest, fun, and other positive affective experiences that students derive from engaging in physical activity (Kendzierski and DeCarlo, 1991; Chen et al., 2021). Rather than indexing physical activity frequency, physical competence, or instrumental beliefs about health benefits, PAE focuses primarily on the affective quality of the activity experience itself. Cognitive evaluation theory proposes that social-contextual conditions that support autonomy and competence can facilitate intrinsic motivation and sustained interest (Deci and Ryan, 1985). In PE settings, when teachers offer meaningful choices, acknowledge students’ perspectives and feelings, provide encouragement and informational feedback, and explain the rationale and value of physical activity, students may be more likely to experience participation as self-endorsed and personally meaningful rather than externally imposed. Wang and Liu (2007) found that relative autonomy, task orientation, and perceived competence were positively associated with enjoyment in PE among female secondary school students. Mandigo et al. (2008) also documented favorable motivational experiences, including enjoyment, among students who participated in games lessons delivered using autonomy-supportive strategies. More direct intervention evidence indicated that autonomy-supportive PE instruction was accompanied by greater increases in satisfaction with engaging in physical activity than a comparison condition (Fin et al., 2019). Although these studies focused primarily on enjoyment in PE, their findings are conceptually relevant to the broader PAE construct examined in the present study. Together, these theoretical and empirical findings suggest that students who perceive greater autonomy support from their PE teachers may report higher levels of PAE.

PAE may, in turn, be associated with IPA. The theory of planned behavior proposes that behavioral intentions are informed by attitudes toward the behavior, subjective norms, and perceived behavioral control (Ajzen, 1991). Although PAE is not identical to the attitude construct specified in the theory of planned behavior, it is conceptually related to affective evaluations of physical activity and may contribute to more favorable attitudes toward future participation. When students experience physical activity as enjoyable, interesting, and worthwhile, they may be more likely to form stronger intentions to remain physically active. Systematic review evidence has highlighted the relevance of autonomous forms of motivation, particularly intrinsic motivation, to the adoption and maintenance of exercise and physical activity (Teixeira et al., 2012). Meta-analytic evidence has also shown that affective judgments, including enjoyment and liking, are positively associated with physical activity among young people (Nasuti and Rhodes, 2013). More directly, Cuevas-Campos et al. (2020) reported a positive association between enjoyment in PE and intention to be physically active among adolescents. Thus, PAE may represent a theoretically plausible affective pathway through which PPETAS is indirectly associated with IPA. Based on this theoretical and empirical rationale, the following hypothesis was proposed:

*H2*: Perceived PE teacher autonomy support will be indirectly associated with intention to be physically active through physical activity enjoyment.

### 2.3 Hypothesized indirect association through exercise identity

Exercise identity refers to the extent to which the role of being an exerciser is incorporated into and becomes salient within an individual’s self-concept (Anderson and Cychosz, 1994). Whereas PAE primarily concerns the affective quality of participation, EI concerns whether exercise is represented in students’ self-definition. Organismic integration theory explains how externally prompted behaviors and their underlying values may become progressively internalized and integrated with the self when the social context supports autonomy (Deci and Ryan, 2000). In PE settings, teachers who offer meaningful choices, explain the rationale and value of physical activity, acknowledge students’ perspectives and feelings, and provide informational feedback may create conditions in which students more fully endorse the personal value of exercise. Such endorsement may be conceptually relevant to incorporating exercise into the self-concept. Direct evidence linking PPETAS with EI appears limited. Among adult exercise participants, Vlachopoulos et al. (2011) found that exercise role identity was associated with competence need satisfaction and more autonomous forms of behavioral regulation; however, perceived autonomy support from exercise instructors did not explain additional variance after these motivational variables were taken into account. Strachan et al. (2013) likewise reported strong positive associations between exercise-identity strength and identified, integrated, and intrinsic regulations. In school PE, meta-analytic evidence has shown that psychological need satisfaction is closely related to autonomous motivation and that autonomous motivation is positively associated with adaptive outcomes, although EI was not directly assessed (Vasconcellos et al., 2020). Collectively, this evidence provides indirect, theory-consistent support for the possibility that students who perceive greater autonomy support from their PE teachers may report a stronger EI.

Exercise identity may, in turn, be associated with IPA. Within identity theory, role identities comprise role-based meanings attached to the self; when salient, they may organize intentions and behavior toward consistency with and verification of those identity meanings (Stets and Burke, 2000). In the physical activity context, students who regard being an exerciser as part of who they are may be more likely to form intentions consistent with that self-definition, including intentions to remain physically active outside PE classes and after graduation. Conceptually related empirical findings support this proposition. In adolescent samples from Estonia and Spain, physical activity self-identity was positively associated with physical activity intention and behavior (Ries et al., 2012). A review and meta-analysis of physical activity identity and schema research also identified intention as one of the psychological correlates and potential pathways linking identity with physical activity (Rhodes et al., 2016). Thus, EI may represent a theoretically plausible identity-based pathway through which PPETAS is indirectly associated with IPA. Based on this theoretical and empirical rationale, the following hypothesis was proposed:

*H3*: Perceived PE teacher autonomy support will be indirectly associated with intention to be physically active through exercise identity.

### 2.4 Hypothesized serial indirect association involving physical activity enjoyment and exercise identity

Building on the preceding arguments, PAE and EI may not only represent separate statistical indirect pathways linking PPETAS with IPA but may also be related in a theoretically specified sequence. PAE captures the pleasure, interest, and positive affective quality of students’ participation in physical activity, whereas EI reflects the extent to which exercise is incorporated into their self-concept. The physical activity self-definition model proposes that enjoyment, perceived wanting, perceived trying, perceived commitment, and perceived ability contribute directly or indirectly to the extent to which individuals define themselves as physically active (Kendzierski and Morganstein, 2009). Importantly, the model does not posit a simple direct link between enjoyment and physical activity self-definition. Rather, it situates enjoyment within a broader set of experiential and self-regulatory processes associated with physical activity self-definition. Consistent with this broader proposition, a review and meta-analysis of physical activity identity and schema research found positive associations between affective judgments, including enjoyment, and physical activity identity or schema, while emphasizing that the available evidence was predominantly correlational (Rhodes et al., 2016). Positive associations between physical activity enjoyment and dimensions of physical self-concept have also been reported among schoolchildren (Lohbeck et al., 2016). Moreover, a 16-week intervention study among low-active adults provided partial support for the physical activity self-definition model, in which enjoyment was specified as an indirect correlate of physical activity self-definition (Morgan et al., 2021). Because these studies examined different constructs and populations and did not establish a direct temporal PAE-to-EI process, they provide only indirect support for the proposed ordering. Accordingly, the PAE-to-EI link was treated as a theory-specified but exploratory component of the serial model.

Taken together, these complementary theoretical perspectives provide a rationale for the hypothesized-order model. Cognitive evaluation theory supports the proposed association between PPETAS and PAE, as autonomy-supportive PE environments may support students’ interest, enjoyment, and positive affective experiences in physical activity (Deci and Ryan, 1985). The physical activity self-definition model offers a broader conceptual rationale for linking positive physical activity experiences with exercise-related self-definition, whereas identity theory suggests that stronger EI may be associated with intentions consistent with that self-definition (Stets and Burke, 2000). Accordingly, the hypothesized-order model specified PAE as the first mediator and EI as the second. This ordering was specified *a priori*, whereas the reversed EI-to-PAE ordering was evaluated as an alternative statistical specification. Because the data were cross-sectional, neither ordering was interpreted as evidence of a temporal or causal sequence. Based on this theory-consistent but indirect evidence, the following model-specified hypothesis was proposed:

*H4*: In the hypothesized-order model, perceived PE teacher autonomy support will show a serial indirect association with intention to be physically active through physical activity enjoyment followed by exercise identity.

Taken together, the reviewed theory and evidence provide a rationale for testing the expected positive association between PPETAS and IPA, two specific indirect associations through PAE and EI, and a model-specified serial indirect association through PAE followed by EI. Figure 1 presents the integrated conceptual framework and theoretical rationale. Specifically, the study examined (1) the positive association between PPETAS and IPA; (2) the specific indirect association through PAE; (3) the specific indirect association through EI; and (4) the hypothesized-order serial indirect association through PAE followed by EI.

**Figure 1:**
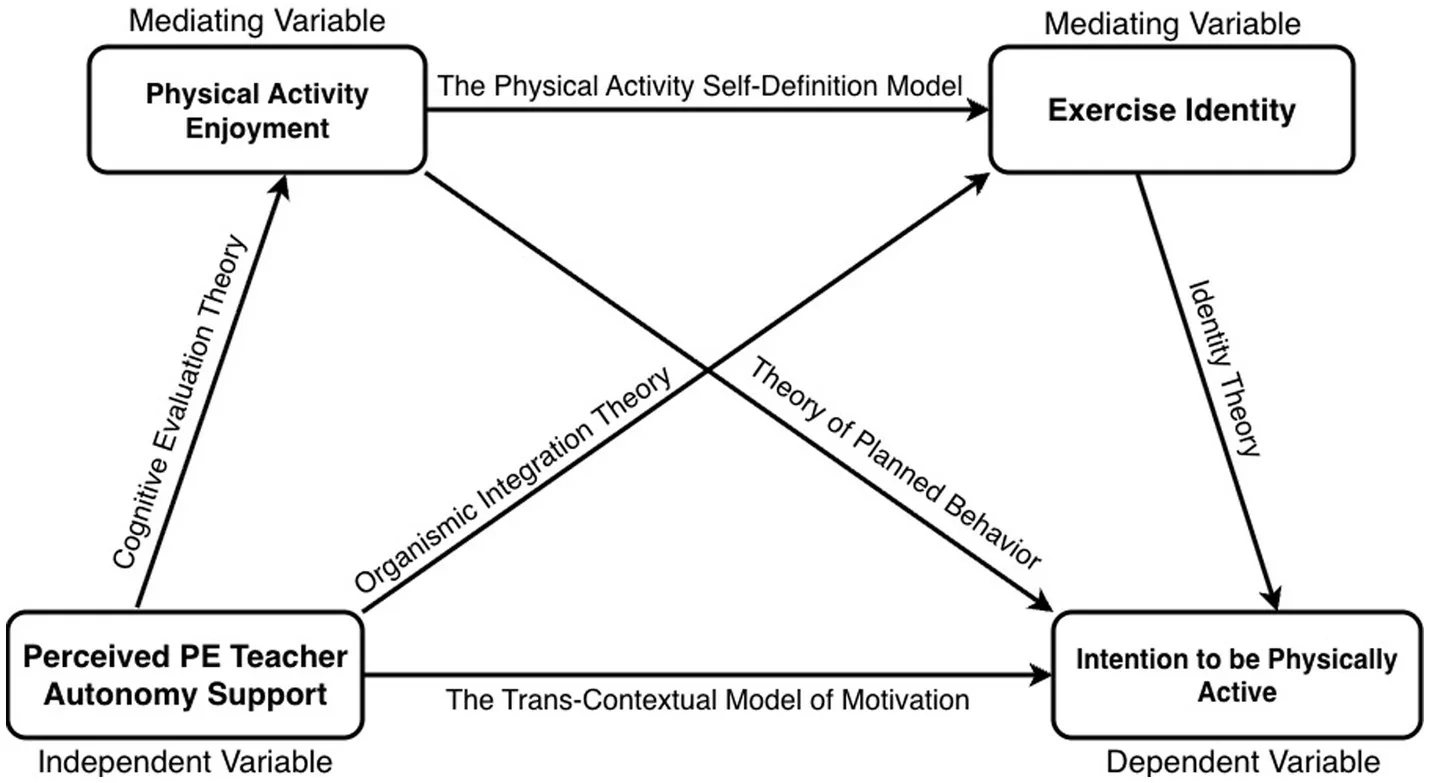
Integrated conceptual framework and theoretical rationale for the hypothesized associations among perceived physical education teacher autonomy support, physical activity enjoyment, exercise identity, and intention to be physically active.

## 3 Materials and methods

### 3.1 Participants and procedure

This cross-sectional questionnaire survey was conducted at four general senior high schools in Luoyang, Henan Province, China. Each school organized one scheduled day of questionnaire administration. The four school-specific administrations and initial data-quality screening were completed between June 25 and July 4, 2026. Administrative permission was obtained from all four participating schools, and homeroom and subject teachers assisted with survey coordination and questionnaire administration. Enrolled students were then invited to participate voluntarily. Data were collected using paper-and-pencil and online questionnaires with identical items, instructions, and response options. Paper questionnaires were administered in person, whereas online questionnaires were completed through the Wenjuanxing platform. Because the national college entrance examination (gaokao) had concluded, all retained Grade 12 respondents (*n* = 553) received the online questionnaire link through class-group messaging channels coordinated by their homeroom teachers. Administration mode was not retained for the remaining respondents in the anonymized analytic dataset; therefore, complete mode-specific counts could not be reconstructed retrospectively. A total of 2,000 questionnaires were distributed. Following data-quality screening, 1,824 responses were retained for analysis, yielding a usable-response proportion of 91.2% among the distributed questionnaires. Responses were excluded if the questionnaire was incomplete, if substantial missing data on core-variable items prevented the calculation of scale scores, or if the demographic information or response patterns showed clear logical inconsistencies. The final analytic sample comprised 829 male students (45.4%) and 995 female students (54.6%). Of the participants, 460 (25.2%) were aged 15 years or younger, 648 (35.5%) were aged 16 years, 511 (28.0%) were aged 17 years, and 205 (11.2%) were aged 18 years or older. By grade level, 682 students (37.4%) were in Grade 10, 589 (32.3%) were in Grade 11, and 553 (30.3%) were in Grade 12. In China, senior high school comprises Grades 10–12, with Grade 12 representing the final year. Grade 12 students remained formally enrolled at the time of participation; for these students, “after graduation” referred to the imminent period following senior high school graduation. The study protocol was reviewed and approved by the institutional ethics review body under the Academic Committee of Luoyang Vocational College of Culture and Tourism (approval no. LYVCCT-LLSC001). Before questionnaire administration, the research team informed students of the study purpose, questionnaire content, voluntary nature of participation, intended use of the data, and their right to decline or withdraw. For participants younger than 18 years, informed consent was obtained from a parent or legal guardian, and assent was obtained from the student. Participants aged 18 years or older provided their own informed consent. Participation was voluntary for all students. The questionnaires were completed anonymously, and all data were used solely for academic research.

### 3.2 Measures

All four measures were administered in Chinese using study-specific translations of the original English items. The translations were intended to preserve the original item meanings and scoring directions, and their psychometric performance in the present sample was evaluated through item-distribution analyses, confirmatory factor analysis (CFA), internal consistency, convergent validity, and discriminant validity.

#### 3.2.1 Perceived PE teacher autonomy support

PPETAS was assessed using the 12-item PE-teacher version of the Perceived Autonomy Support Scale for Exercise Settings (PASSES; Hagger et al., 2007). The scale was developed from earlier autonomy-support measures and behavioral indicators used in educational settings (Black and Deci, 2000; Reeve and Jang, 2006) and assesses the extent to which students perceive that their PE teachers support participation in active sports and/or vigorous exercise during free time. A sample item is, “My PE teacher encourages me to do active sports and/or vigorous exercise in my free time.” The original seven-point response format was adapted to five points, ranging from 1 (not at all true) to 5 (completely true), while item content and scoring direction were retained. All items were scored in the same direction, and higher mean scores indicated greater PPETAS. Cronbach’s *α* was 0.927, and the one-factor CFA showed good fit, *χ*^2^(54) = 161.552, *p* < 0.001, CFI = 0.990, TLI = 0.988, RMSEA = 0.033, 90% CI [0.027, 0.039]. Item distributions and complete measurement results are reported in Supplementary Tables S1–S3.

#### 3.2.2 Physical activity enjoyment

PAE was assessed using the four-item Short Version of the Physical Activity Enjoyment Scale (PACES-S; Chen et al., 2021), which was developed from earlier versions of the Physical Activity Enjoyment Scale (Kendzierski and DeCarlo, 1991; Motl et al., 2001). The unidimensional scale assesses pleasure, enjoyment, and positive affective experiences during physical activity. A sample item is, “I enjoy it.” Responses were recorded on a five-point scale ranging from 1 (not at all true) to 5 (completely true). All items were scored in the same direction, and higher mean scores indicated greater PAE. Cronbach’s *α* was 0.800, and the one-factor CFA showed good fit, *χ*^2^(2) = 3.577, *p* = 0.167, CFI = 0.999, TLI = 0.998, RMSEA = 0.021, 90% CI [0.000, 0.055]. Complete measurement results are reported in Supplementary Tables S2, S3.

#### 3.2.3 Exercise identity

EI was assessed using the nine-item Exercise Identity Scale (EIS; Anderson and Cychosz, 1994; Wilson and Muon, 2008), which measures the extent to which exercise is incorporated into an individual’s self-concept. A sample item is, “I consider myself an exerciser.” The original seven-point response format was adapted to five points, ranging from 1 (not at all true) to 5 (completely true), while item content and scoring direction were retained. All items were scored in the same direction, and higher mean scores indicated stronger EI. Cronbach’s *α* was 0.910. The one-factor model showed good fit, *χ*^2^(27) = 46.754, *p* = 0.011, CFI = 0.998, TLI = 0.997, RMSEA = 0.020, 90% CI [0.010, 0.029]. An alternative two-factor model representing role identity and exercise beliefs also fit well, *χ*^2^(26) = 46.589, *p* = 0.008, CFI = 0.997, TLI = 0.996, RMSEA = 0.021, 90% CI [0.011, 0.030], but provided no meaningful improvement and the two factors correlated at 0.997. The more parsimonious one-factor representation was therefore retained. Item distributions and complete model comparisons are reported in Supplementary Tables S1–S3.

#### 3.2.4 Intention to be physically active

IPA was assessed using the four-item Intention to Be Physically Active After Graduation Questionnaire (Hein et al., 2004; Erdvik et al., 2015), which assesses students’ intentions to remain physically active after graduation. During scale development, Hein et al. (2004) removed the item “I often do sport in my free time” because of its comparatively low loading and content-validity concerns, yielding the four-item form subsequently translated and validated by Erdvik et al. (2015). A sample item is, “After graduation, I would like to be physically active.” Responses were recorded on a five-point scale, consistent with the number of response categories used by Hein et al. (2004), with harmonized anchors ranging from 1 (not at all true) to 5 (completely true). All items were scored in the same direction, and higher mean scores indicated stronger IPA. Cronbach’s *α* was 0.814, and the one-factor CFA showed good fit, *χ*^2^(2) = 5.328, *p* = 0.070, CFI = 0.999, TLI = 0.996, RMSEA = 0.030, 90% CI [0.000, 0.063]. Complete measurement results are reported in Supplementary Tables S2, S3.

### 3.3 Statistical analysis

Statistical analyses used SPSS 26.0, AMOS 26.0, and the PROCESS macro (version 4.1). Descriptive statistics, distributions of the two rescaled scales, and Cronbach’s *α* were examined. The retained analytic sample had complete data on all 29 focal items and covariates; therefore, no imputation was required. Univariate and multivariate distributional assumptions were examined using item skewness, kurtosis, and multivariate kurtosis before the latent-variable analyses. Harman’s test and a 29-item single-factor CFA were used as preliminary common-method diagnostics. CFA evaluated the individual-scale models, the one- and two-factor EIS models, and the correlated four-construct model using *χ*^2^, df, *p*, CFI, TLI, RMSEA with its 90% confidence interval, and SRMR. Standardized loadings, composite reliability (CR), average variance extracted (AVE), and the Fornell–Larcker criterion assessed convergent and discriminant validity. No correlated item residuals or modification-index-based respecifications were used. Composite scores were used for descriptive statistics and Pearson correlations. The primary analysis used maximum-likelihood latent-variable structural equation modeling (SEM), with all 29 items specified as indicators of their respective constructs. Group means were reported descriptively because measurement invariance was not tested. The hypothesized-order model placed PAE before EI, whereas a reversed-order latent-variable model placed EI before PAE and was evaluated as an alternative statistical specification. A composite-score PROCESS Model 6 analysis, controlling for gender, age, and grade, was used as a robustness check, with specific indirect effects evaluated within the full model. Multicollinearity in the composite-score robustness analysis was assessed using tolerance and variance inflation factors. Indirect effects were estimated with 5,000 resamples and 95% percentile bootstrap CIs; intervals excluding zero indicated nonzero indirect effects. Other tests were two-tailed at *p* < 0.05. Model orderings were interpreted as cross-sectional statistical specifications rather than temporal or causal sequences.

## 4 Results

### 4.1 Descriptive statistics of perceived PE teacher autonomy support, physical activity enjoyment, exercise identity, and intention to be physically active

Table 1 presents the overall mean scores for the four study variables: perceived PE teacher autonomy support (*M* = 3.15, SD = 0.54), physical activity enjoyment (*M* = 3.45, SD = 0.57), exercise identity (*M* = 3.35, SD = 0.55), and intention to be physically active (*M* = 3.50, SD = 0.58). Male and female subgroup means are also presented descriptively in Table 1.

**Table 1:** Descriptive statistics (*M* ± SD) for the study variables.

Group	N	Perceived PE teacher autonomy support	Physical activity enjoyment	Exercise identity	Intention to be physically active
Male	829	3.18 ± 0.55	3.49 ± 0.59	3.41 ± 0.56	3.55 ± 0.57
Female	995	3.13 ± 0.53	3.41 ± 0.55	3.30 ± 0.55	3.45 ± 0.58
Overall	1,824	3.15 ± 0.54	3.45 ± 0.57	3.35 ± 0.55	3.50 ± 0.58

Values are presented as *M* ± SD. Male and female subgroup statistics are reported descriptively; no inferential group comparisons are made because measurement invariance across gender was not evaluated. Demographic distributions by age and grade are reported in the Participants section.

### 4.2 Assessment of common method bias

Harman’s single-factor test and a 29-item single-factor CFA were used as preliminary common-method diagnostics. The unrotated principal component solution yielded four components with eigenvalues greater than 1, with the first accounting for 35.44% of the total variance. The single-factor CFA fit the data poorly, *χ*^2^(377) = 8,529.629, *p* < 0.001, CFI = 0.674, TLI = 0.648, RMSEA = 0.109, 90% CI [0.107, 0.111], compared with the correlated four-construct model, *χ*^2^(371) = 477.151, *p* < 0.001, CFI = 0.996, TLI = 0.995, RMSEA = 0.013, 90% CI [0.009, 0.016], SRMR = 0.016. These results did not support a dominant single-factor account, although common method bias could not be ruled out.

### 4.3 Correlation analysis of perceived PE teacher autonomy support, physical activity enjoyment, exercise identity, and intention to be physically active

Table 2 presents positive Pearson correlations among the composite scores for the four study variables. Specifically, perceived PE teacher autonomy support was positively correlated with intention to be physically active (*r* = 0.426, *p* < 0.001), physical activity enjoyment (*r* = 0.348, *p* < 0.001), and exercise identity (*r* = 0.489, *p* < 0.001). Intention to be physically active was also positively correlated with physical activity enjoyment (*r* = 0.342, *p* < 0.001) and exercise identity (*r* = 0.397, *p* < 0.001), and physical activity enjoyment was positively correlated with exercise identity (*r* = 0.404, *p* < 0.001). These bivariate associations provided descriptive context for the hypothesized-order latent-variable SEM and the composite-score PROCESS Model 6 robustness analysis.

**Table 2:** Means, standard deviations, and correlations among study variables.

Variable	*M*	SD	1	2	3	4
Perceived PE teacher autonomy support	3.15	0.54	1			
Physical activity enjoyment	3.45	0.57	0.348^***^	1		
Exercise identity	3.35	0.55	0.489^***^	0.404^***^	1	
Intention to be physically active	3.50	0.58	0.426^***^	0.342^***^	0.397^***^	1

*N* = 1,824. *M*, mean; SD, standard deviation. Pearson correlations among the study variables are reported. PPETAS, Perceived PE Teacher Autonomy Support; PAE, Physical Activity Enjoyment; EI, Exercise Identity; IPA, Intention to be Physically Active. ^***^p < 0.001.

### 4.4 Latent-variable SEM and robustness analyses

The correlated four-construct measurement model showed good fit, *χ*^2^(371) = 477.151, *p* < 0.001, CFI = 0.996, TLI = 0.995, RMSEA = 0.013, 90% CI [0.009, 0.016], and SRMR = 0.016. Standardized loadings ranged from 0.680 to 0.760, CR values from 0.800 to 0.927, and AVE values from 0.500 to 0.528; the Fornell–Larcker criterion was satisfied. Complete measurement results are reported in Supplementary Tables S2, S3. The hypothesized-order latent-variable SEM (PPETAS → PAE → EI → IPA) showed the same fit as the correlated measurement model and is presented in Figure 2. The composite-score PROCESS Model 6 analysis, controlling for gender, age, and grade, was retained as a robustness analysis (Tables 3, 4), and complete latent-variable estimates are provided in Supplementary Table S4. PPETAS was positively associated with PAE (*β* = 0.405), EI (*β* = 0.409), and IPA (*β* = 0.295); PAE was positively associated with EI (*β* = 0.307) and IPA (*β* = 0.207); and EI was positively associated with IPA (*β* = 0.204), all *p* < 0.001. The model explained 16.4, 36.3, and 32.6% of the variance in PAE, EI, and IPA, respectively. With 5,000 percentile bootstrap resamples, the specific indirect associations through PAE (*β* = 0.084, 95% CI [0.058, 0.111]), through EI (*β* = 0.084, 95% CI [0.058, 0.111]), and through PAE followed by EI (*β* = 0.025, 95% CI [0.017, 0.035]) all had confidence intervals excluding zero. The standardized direct association, total indirect association, and total association were 0.295, 0.193, and 0.488, respectively. The positive total association was consistent with H1, the specific indirect associations through PAE and EI were consistent with H2 and H3, and the hypothesized-order serial association was consistent with the model-specified H4. The reversed-order model (PPETAS → EI → PAE → IPA) showed identical fit. Its specific indirect associations through PAE (*β* = 0.044, 95% CI [0.028, 0.063]), through EI (*β* = 0.109, 95% CI [0.076, 0.143]), and through EI followed by PAE (*β* = 0.040, 95% CI [0.027, 0.054]) also had confidence intervals excluding zero. Thus, the cross-sectional data were compatible with both mediator orderings and did not identify a unique temporal sequence. The composite-score PROCESS Model 6 robustness analysis yielded a consistent pattern of direct and specific indirect associations (Tables 3, 4).

**Figure 2:**
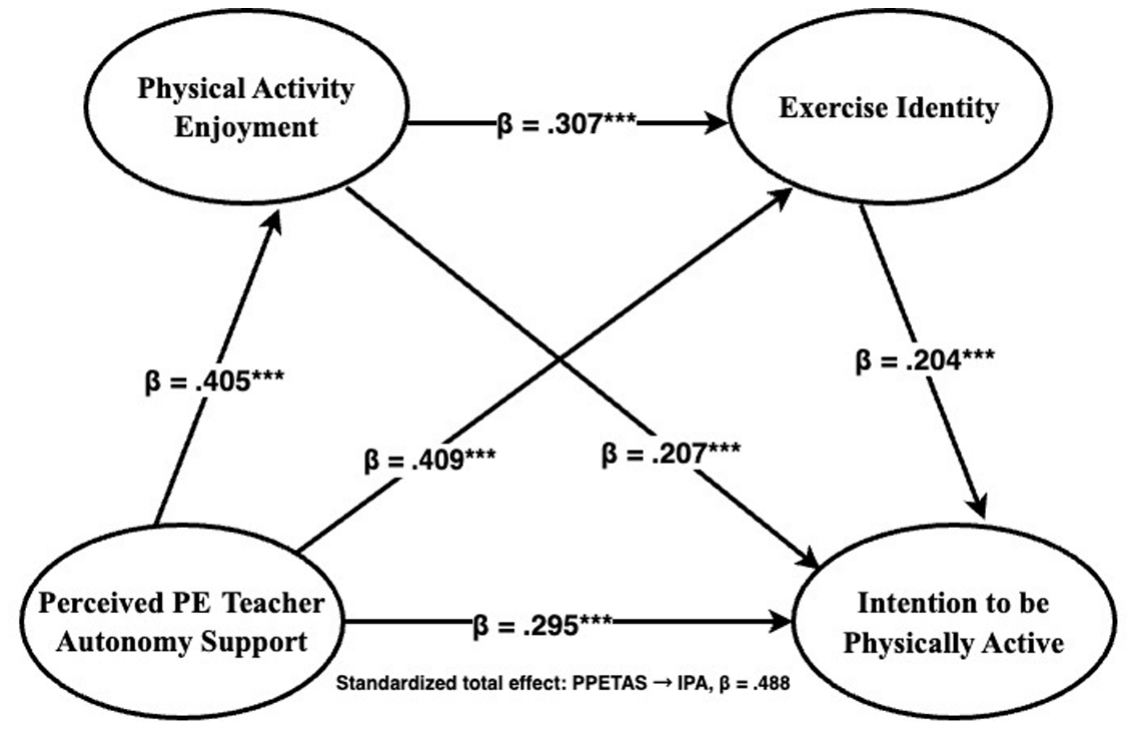
Standardized results for the hypothesized-order latent-variable structural equation model. *N* = 1,824. Values shown on the arrows are standardized structural coefficients (*β*) from the hypothesized-order latent-variable structural equation model. PPETAS, perceived PE teacher autonomy support; PAE, physical activity enjoyment; EI, exercise identity; IPA, intention to be physically active. The coefficient shown on the PPETAS → IPA path represents the standardized direct effect after PAE and EI were included in the model (*β* = 0.295), whereas the standardized total effect was *β* = 0.488. The model explained 16.4%, 36.3%, and 32.6% of the variance in PAE, EI, and IPA, respectively. Model fit: *χ*²(371) = 477.151, *p* < 0.001, CFI = 0.996, TLI = 0.995, RMSEA = 0.013, 90% CI [0.009, 0.016], and SRMR = .016. Specific indirect effects and their 95% percentile bootstrap confidence intervals are reported in Supplementary Table S4. ^***^p < 0.001.

**Table 3:** Regression analysis of the serial indirect-effects model (composite-score PROCESS Model 6 robustness analysis).

Variable	Physical activity enjoyment		Exercise identity		Intention to be physically active		Total-effect model	
	*β*	*t*	*β*	*t*	*β*	*t*	*β*	*t*
Perceived PE teacher autonomy support	0.341	15.275^***^	0.386	18.286^***^	0.263	11.029^***^	0.409	19.024^***^
Physical activity enjoyment	–	–	0.260	12.487^***^	0.167	7.424^***^	–	–
Exercise identity	–	–	–	–	0.187	7.691^***^	–	–
*R* ^2^	0.125		0.308		0.257		0.192	
*F*	65.079^***^		162.118^***^		104.597^***^		107.861^***^	

Standardized coefficients (*β*) are reported. Results are from the composite-score PROCESS Model 6 robustness analysis. Gender, age, and grade were included as covariates in all equations but are not shown. Dashes indicate paths that were not estimated in the corresponding equation. t values are reported as absolute values. The *F* row reports model *F* statistics. ^***^p < 0.001.

**Table 4:** Indirect, direct, and total effects from the composite-score PROCESS Model 6 robustness analysis.

Path	Unstandardized effect (*B*)	95% CI	
		LL	UL
PPETAS → PAE → IPA	0.061	0.0431	0.0793
PPETAS → EI → IPA	0.077	0.0555	0.0994
PPETAS → PAE → EI → IPA	0.018	0.0122	0.0235
Total indirect effect	0.155	0.1273	0.1837
Direct effect (*c*′)	0.280	0.2298	0.3292
Total effect (*c*)	0.435	0.3899	0.4796

*N* = 1,824. Effects are unstandardized (*B*). Specific and total indirect effects were estimated using 5,000 percentile bootstrap resamples; confidence intervals for the direct and total effects are conventional 95% confidence intervals. Gender, age, and grade were covariates. Completely standardized indirect effects were 0.146 [0.1205, 0.1718] in total, 0.057 [0.0408, 0.0744] via PAE, 0.072 [0.0521, 0.0931] via EI, and 0.017 [0.0115, 0.0221] via PAE followed by EI. PPETAS, perceived PE teacher autonomy support; PAE, physical activity enjoyment; EI, exercise identity; IPA, intention to be physically active.

## 5 Discussion

This study used latent-variable SEM as the primary analysis to examine the association between PPETAS and IPA and the specific and serial indirect associations involving PAE and EI among Chinese senior high school students. In the hypothesized-order model, PPETAS showed positive total and direct associations with IPA, and the specific indirect associations through PAE and EI and the serial indirect association through PAE followed by EI were all nonzero. The reversed-order model also yielded a nonzero serial indirect association, and the composite-score PROCESS Model 6 robustness analysis produced a directionally consistent pattern for the hypothesized ordering. These findings suggest that students’ future intentions to be physically active may be related to PPETAS, physical activity enjoyment, and exercise identity. Because both mediator orderings were compatible with the cross-sectional data, the findings do not identify a unique temporal sequence or establish causal mechanisms; moreover, the outcome was intention rather than actual physical activity behavior.

### 5.1 Perceived PE teacher autonomy support and intention to be physically active

The hypothesized-order latent-variable SEM showed a positive total association between PPETAS and IPA, and the direct association remained positive after PAE and EI were included, consistent with H1. This finding is consistent with previous research in PE contexts, which has generally indicated that students who perceive greater autonomy support from their PE teachers tend to report stronger physical activity intentions. Previous research showed that PE teacher autonomy support may be associated with students’ future physical activity intentions through basic psychological need satisfaction and autonomous motivation (Leyton-Román et al., 2020). Need-supportive teaching in PE was also positively associated with the satisfaction of basic psychological needs and novelty, which were, in turn, positively associated with intention to be physically active (Aibar et al., 2021). In addition, longitudinal research based on the trans-contextual model of motivation provided further support for a cross-contextual motivational process in which perceived autonomy support from PE teachers was associated with autonomous motivation in PE, which was subsequently associated with autonomous motivation in leisure-time physical activity and with physical activity intention through related motivational processes (Kalajas-Tilga et al., 2022). By examining this association among Chinese senior high school students, the present study extends evidence on PPETAS and future physical activity intentions to this educational and cultural context.

The trans-contextual model provides a relevant broader framework for interpreting this association. It proposes that autonomy support in PE may relate to out-of-school physical activity intentions through autonomous motivation across PE and leisure-time contexts; because these intervening motivational constructs were not assessed, the present findings should not be regarded as a test of the full model (Hagger et al., 2003; Hagger and Chatzisarantis, 2016). By offering meaningful choices, explaining the rationale for activities, acknowledging students’ feelings, providing informational feedback, and encouraging autonomous participation, PE teachers may help students view physical activity as personally meaningful and worth sustaining over time. Research involving Chinese students in PE has also shown that perceived autonomy support is associated with psychological need satisfaction, subjective vitality, and effort (Taylor and Lonsdale, 2010). In addition, perceived autonomy support in PE was positively associated with autonomous motivation toward leisure-time physical activity among Chinese college students (Abula et al., 2020). Students’ physical activity intentions may also be associated with autonomy support from peers and parents, although the contributions of these sources are not necessarily equivalent (Hagger et al., 2009). More broadly, research in online learning has shown that the association between social support and learning burnout may vary with students’ self-regulated learning, although this evidence comes from a different educational context and does not directly address PPETAS or physical activity intentions (Huang et al., 2025). Broader youth-sport research has also linked self-esteem and perceived social support to wellbeing among young athletes and suggested that physical activity may buffer social-anxiety and sleep-related risks among young e-sports players, although these studies did not assess PPETAS, PAE, EI, or IPA (Solmaz, 2025; Solmaz et al., 2025). Taken together, the present finding extends evidence in Chinese senior high school PE, while longitudinal, intervention, and multilevel studies are needed to evaluate temporal and contextual processes.

### 5.2 Specific indirect association through physical activity enjoyment

In the full hypothesized-order latent-variable SEM, with EI included simultaneously, the specific indirect association between PPETAS and IPA through PAE was nonzero, consistent with H2. This finding suggests that students’ perceptions of PE teacher autonomy support are related not only to their future intentions to be physically active but also to the enjoyment, interest, and positive feelings they experience during physical activity. Previous research has shown a positive association between autonomy-supportive PE teaching and students’ satisfaction and enjoyment. Specifically, Baños et al. (2020) found that satisfaction/enjoyment in PE mediated the association between perceived autonomy support and academic performance in PE. Similarly, students reported greater enjoyment and perceived competence during a hybrid teaching games for understanding and sport education unit than during traditional direct instruction (Gil-Arias et al., 2020). Regarding physical activity intentions, enjoyment in PE was positively associated with intention to be physically active (Cuevas-Campos et al., 2020), and enjoyment was also among the achievement emotions positively associated with students’ future intention to remain physically active (Fierro-Suero et al., 2023). By integrating evidence on autonomy support and enjoyment with evidence on enjoyment and intention in a Chinese senior high school sample, the present study suggests that PAE may represent a distinct affective correlate within the full model of the PPETAS–IPA association.

From a theoretical perspective, this pattern is consistent with cognitive evaluation theory (Deci and Ryan, 1985) and the theory of planned behavior (Ajzen, 1991). Cognitive evaluation theory proposes that social contexts supporting autonomy and competence can sustain interest and intrinsic motivation. In PE settings, when teachers offer meaningful choices, explain the rationale for activities, acknowledge students’ feelings, provide informational feedback, and encourage autonomous participation, students may be more likely to experience physical activity as self-endorsed rather than externally imposed and may consequently report greater physical activity enjoyment. From the perspective of the theory of planned behavior, physical activity enjoyment is conceptually related to a favorable affective evaluation of physical activity, although it is not identical to the attitude construct specified by the theory. Such affective evaluations may be associated with stronger intentions to participate in physical activity. Broader physical activity research has also demonstrated the relevance of affective attitudes to physical activity intention and behavior (Phipps et al., 2021), while longitudinal research in PE has identified associations among motivational climate, motivational regulations, enjoyment, and physical activity participation (Jaakkola et al., 2017). For Chinese senior high school students, enjoyable physical activity experiences may therefore represent an affective correlate of intentions to remain physically active, alongside perceived PE teacher autonomy support.

### 5.3 Specific indirect association through exercise identity

In the full hypothesized-order latent-variable SEM, with PAE included simultaneously, the specific indirect association between PPETAS and IPA through EI was nonzero, consistent with H3. This finding suggests that the association between students’ perceptions of PE teacher autonomy support and their future intentions to be physically active may extend beyond external support and immediate activity experiences to whether exercise is incorporated into their self-concept. Previous research has documented a relatively consistent positive association between physical activity identity or self-schema and physical activity behavior (Rhodes et al., 2016). In adolescent samples, physical activity self-identity was positively associated with physical activity intention and with physical activity behavior assessed 4 weeks later (Ries et al., 2012). Further longitudinal research showed that exercise identity prospectively predicted subsequent physical activity during late adolescence, suggesting that exercise identity may be an important psychological factor in adolescents’ physical activity development (Porter et al., 2024). In addition, research integrating exercise self-identity with the theory of planned behavior indicated that self-identity contributed to the prediction of exercise behavior and interacted with exercise intention in predicting behavior (de Bruijn and van den Putte, 2012). Because much of this evidence concerns actual physical activity behavior rather than IPA, it provides broader contextual rather than directly comparable support. Within the present full model, EI may therefore represent a distinct identity-based correlate of the PPETAS–IPA association.

From a theoretical perspective, this pattern is consistent with organismic integration theory (Deci and Ryan, 1985, 2000) and identity theory (Stets and Burke, 2000). Organismic integration theory describes how autonomy-supportive social contexts may facilitate the internalization and integration of values underlying behavior into the self. In PE settings, when teachers offer meaningful choices, explain the value of physical activity, acknowledge students’ feelings, and provide encouragement, students may be more likely to view exercise as personally valuable for their growth, competence development, and a healthy lifestyle. A systematic review and meta-analysis of self-determination theory–based instructional interventions reported small positive effects on intrinsic motivation and identified regulation (Manninen et al., 2022). Although exercise identity was not assessed in that review, these findings provide indirect support for the internalization rationale linking perceived PE teacher autonomy support with exercise identity. Qualitative research has also suggested that satisfaction of basic psychological needs and sustained participation in physical activity may contribute to the development of a physically active self (Springer et al., 2013). From the perspective of identity theory, when students incorporate the identity of being an exerciser into their self-concept, intending to remain physically active in the future may be more consistent with their identity meanings. Related prospective research indicated that self-regulatory efficacy statistically mediated the association between exercise identity and subsequent exercise behavior (Strachan et al., 2015). For Chinese senior high school students, EI may provide a self-referential framework associated with future physical activity intentions. Within the cross-sectional model, understanding IPA may therefore require attention not only to enjoyable activity experiences but also to whether exercise is incorporated into students’ self-concept.

### 5.4 Serial indirect associations involving physical activity enjoyment and exercise identity

In the hypothesized-order latent-variable SEM, the serial indirect association between PPETAS and IPA through PAE followed by EI was nonzero (*β* = 0.025, 95% CI [0.017, 0.035]), consistent with the model-specified H4. However, the reversed-order model also yielded a nonzero serial indirect association through EI followed by PAE (*β* = 0.040, 95% CI [0.027, 0.054]). These findings indicate that PAE and EI may jointly characterize the PPETAS–IPA association, but they do not identify one mediator ordering as uniquely compatible with the cross-sectional data. PAE captures the positive affective quality of physical activity, whereas EI reflects exercise-related self-definition. The hypothesized PAE-to-EI ordering was specified *a priori* but treated as exploratory because the physical activity self-definition model places enjoyment within a broader set of experiential and self-regulatory correlates rather than proposing a simple direct temporal path from enjoyment to self-definition (Kendzierski and Morganstein, 2009). Existing longitudinal evidence also suggests complex or potentially reciprocal relations among enjoyment, physical activity, and physical self-concept, while intervention evidence indicates that the conceptually related construct of physical activity identity can change over time (Garn et al., 2019; Kruk et al., 2018; Rhodes et al., 2025). Accordingly, these studies provide contextual support for interplay between affective experience and self-related constructs, but not direct evidence that PAE temporally precedes EI.

The hypothesized ordering can be interpreted through complementary theoretical perspectives. Cognitive evaluation theory suggests that autonomy- and competence-supportive contexts can sustain intrinsic motivation and interest, providing a rationale for the PPETAS–PAE association (Deci and Ryan, 1985). The physical activity self-definition model provides a broader rationale for relating positive physical activity experiences to self-definition, whereas identity theory suggests that intentions may align with salient exercise-related identity meanings (Kendzierski and Morganstein, 2009; Stets and Burke, 2000). Existing evidence is also compatible with the reversed ordering. Exercise-identity strength and identity–exercise consistency have been associated with affective responses to identity-consistent or identity-inconsistent exercise, suggesting that EI may shape how physical activity is experienced (Strachan et al., 2009). Research among adults aged 55 years or older has also placed role identity within statistical associations between motivational regulations and physical activity behavior (Amireault and Huffman, 2024). Because these studies involved different populations and behavior outcomes, they provide contextual rather than direct support for the reversed EI-to-PAE specification. The hypothesized-order serial indirect association was smaller than either specific indirect association through PAE or EI alone (*β* = 0.025 vs. 0.084 and 0.084), supporting its interpretation as a supplementary component rather than the primary account of the PPETAS–IPA association. More importantly, the nonzero serial indirect association in the reversed-order model indicates that the cross-sectional data are compatible with both mediator orderings. The contribution of this result therefore lies in identifying the joint statistical relevance of affective experience and exercise-related self-definition, not in establishing a temporal sequence. Because IPA is an intention rather than observed behavior, these associations should not be interpreted as evidence that students will subsequently remain physically active (Rhodes et al., 2022). Longitudinal or multiwave studies are needed to evaluate whether either ordering has temporal validity. Repeated cross-sectional data combined with quasi-experimental analysis can strengthen population-level inference in some settings (Wang et al., 2026), but individual-level multiwave data remain necessary to test the temporal ordering of PAE and EI.

## 6 Practical implications

The findings identify three complementary candidate targets for future PE intervention research: autonomy-supportive teaching, enjoyable and appropriately challenging physical activity experiences, and opportunities for students to reflect on exercise as part of their self-definition. Because both mediator orderings were compatible with the cross-sectional data, these features should be treated as parallel candidate targets rather than components that must be implemented in a fixed PAE-to-EI sequence.

Candidate teacher- and program-level practices could include offering meaningful choices, explaining activity rationales, acknowledging students’ perspectives, using non-controlling language and informational feedback, providing varied activities with adjustable difficulty, and offering goal-setting or progress-feedback opportunities that invite exercise-related self-reflection. These examples are intended to generate testable intervention strategies, not to prescribe an established program.

Future intervention studies should evaluate whether these practices change PAE, EI, and IPA and whether any changes extend to objectively assessed physical activity behavior. The present findings concern intentions and cross-sectional associations; they do not demonstrate that the proposed practices are effective or that enjoyment must precede exercise identity.

## 7 Limitations and future directions

The findings should be interpreted in light of limitations concerning same-source measurement, the cross-sectional design, residual confounding and data nesting, untested measurement invariance, and the geographic and educational concentration of the sample. These limitations also define priorities for future research.

### 7.1 Limitations of measurement methods

The study has several measurement-related limitations. All focal constructs were assessed by student self-report at one time point; PPETAS therefore reflects perceived rather than observed teacher behavior, and the associations may have been affected by social desirability, response-style bias, and common method variance. Harman’s test and the 29-item one-factor CFA were diagnostic checks only and cannot rule out same-source inflation, particularly because no independent marker variable was collected. The study-specific Chinese translations were not subjected to a formal multistep cross-cultural adaptation procedure, and the PPETAS and EI response formats were reduced from seven to five points. Although item-distribution analyses, internal consistency, CFA, CR, AVE, and discriminant validity supported use of the measures in this sample, linguistic equivalence and the extent of any variance compression remain uncertain. Finally, all Grade 12 respondents completed the survey online after the gaokao, whereas administration mode was not retained for the remaining respondents; grade-related and mode-related influences could not be separated. Future studies should use formally adapted translations, direct response-format comparisons, marker-variable or multisource designs, and repeated measurement.

### 7.2 Limitations in study design and causal inference

The cross-sectional design cannot establish temporal precedence or causal direction. The hypothesized PAE-to-EI and reversed EI-to-PAE latent models had identical global fit, and both serial indirect associations were nonzero; therefore, the data do not identify a unique mediator ordering. Reverse or reciprocal relations are also plausible—for example, students with stronger EI or IPA may evaluate their activity experiences or perceived teacher support more favorably. Moreover, the outcome was intention rather than observed physical activity, so the findings do not show that students subsequently remained physically active. Multiwave longitudinal, cross-lagged, and experimental or quasi-experimental designs are needed to test temporal ordering, reciprocal relations, and causal effects.

### 7.3 Limited control of confounding factors and nested data structure

The composite-score robustness analysis adjusted for gender, age, and grade, but potentially important confounders were not measured, including prior physical activity, exercise experience, health status, academic stress, socioeconomic background, parental and peer support, school PE resources, and teacher characteristics. In addition, students were nested within classes and four schools. School and class identifiers were not retained in the anonymized analytic dataset, so intraclass correlation coefficients (ICCs), school fixed effects, and cluster-robust standard errors could not be reconstructed. Ignoring within-cluster dependence may have made conventional standard errors and confidence intervals overly precise. Future studies should retain cluster identifiers, recruit more schools and classes, and evaluate alternative covariate sets using cluster-robust or multilevel models and sensitivity analyses.

### 7.4 Model robustness and measurement validation

The primary latent-variable SEM modeled measurement error, and the reversed mediator ordering was evaluated as an alternative specification. However, the two orderings had identical global fit and both produced nonzero serial indirect associations, so cross-sectional model fit could not adjudicate between them. Other structurally plausible specifications were not exhaustively compared. Measurement invariance across gender, age, and grade was also not tested; accordingly, subgroup means are reported descriptively and no inferential group or structural-path comparisons are made. Dedicated multigroup and longitudinal SEM studies are needed to evaluate measurement invariance, structural stability, and model equivalence.

### 7.5 Sample and generalizability

The sample was drawn from four general senior high schools in one city and cannot be considered nationally representative. Separate counts of eligible, invited, and declining students were not recorded, so participation selection and nonresponse could not be quantified. The findings may not generalize to other provinces, urban or rural schools, vocational schools, other educational stages, out-of-school adolescents, or other cultural contexts. Replication using broader multi-region sampling and complete recruitment-flow records is needed.

### 7.6 Future research directions and applications

Future research should prioritize five directions. First, multiwave longitudinal and cross-lagged designs should compare PAE-before-EI and EI-before-PAE associations over time and examine possible reciprocal relations. Second, randomized or quasi-experimental autonomy-supportive PE interventions should treat PAE and EI as parallel candidate outcomes rather than impose a fixed sequence. Third, self-reports should be combined with teacher observations, behavioral records, and objective physical activity measures to determine whether IPA translates into behavior. Fourth, school, class, and administration-mode identifiers should be retained, and enough higher-level units should be recruited for cluster-robust or multilevel analyses. Fifth, multigroup validation and broader regional replication should precede subgroup or population-level conclusions and the development of larger PE programs.

## 8 Conclusion

This study used latent-variable SEM as the primary analysis to examine the association between PPETAS and IPA and the specific and serial indirect associations involving PAE and EI in a sample of Chinese senior high school students. PPETAS showed positive total and direct associations with IPA, and the specific indirect associations through PAE and EI were nonzero. The hypothesized serial indirect association through PAE followed by EI was also nonzero; however, the reversed EI-to-PAE ordering yielded a nonzero serial indirect association as well. These findings clarify the distinct and joint statistical relevance of physical activity enjoyment and exercise-related self-definition in the PPETAS–IPA association in this sample.

Because both mediator orderings were compatible with the cross-sectional data and the outcome was intention rather than observed physical activity, the findings do not establish a unique temporal sequence or causal mechanism and do not show that students subsequently engaged in physical activity.

## Data Availability

The participant-level dataset is not publicly available because the ethics approval and informed-consent procedures did not include public data deposition, and the approved data-use plan limits dissemination to aggregated results. Aggregated item-level, measurement-model, and structural-model results are provided in the article and Supplementary Tables S1–S4. The AMOS model files and accompanying analysis specifications are provided as supplementary analysis files. The PROCESS robustness analysis was conducted through the SPSS graphical dialog interface, and no standalone saved PROCESS syntax file was generated. Requests concerning access to de-identified participant-level data may be directed to the corresponding author and will be considered subject to institutional and ethical approval.
