# Supplementary material for: Perceived physical education teacher autonomy support and intention to be physically active among Chinese senior high school students: cross-sectional serial indirect associations involving physical activity enjoyment and exercise identity
Source: Front Psychol. 2026 Sep 24;17:1935171. doi: 10.3389/fpsyg.2026.1935171 (PMC13645541; doi:10.3389/fpsyg.2026.1935171)
Supplement: Supplementary file 1 [file Supplementary_File_1.pdf]

Supplementary Material

1 Supplementary Tables

Supplementary Table S1. Item-level distributions for the administered five-point PPETAS and EIS versions

Scale	Item	M	SD	Skewness	Kurtosis	Floor (%)	Ceiling (%)
PPETAS	PPETAS_1	3.15	.734	.016	-.121	0.6	2.3
PPETAS	PPETAS_2	3.15	.732	-.089	.056	1.0	2.0
PPETAS	PPETAS_3	3.15	.741	.069	-.118	0.5	2.7
PPETAS	PPETAS_4	3.15	.734	-.050	-.157	0.7	2.0
PPETAS	PPETAS_5	3.15	.728	-.050	.023	0.9	2.1
PPETAS	PPETAS_6	3.15	.735	-.052	-.040	0.9	2.1
PPETAS	PPETAS_7	3.15	.727	-.032	-.168	0.6	1.9
PPETAS	PPETAS_8	3.15	.736	-.013	-.287	0.5	2.0
PPETAS	PPETAS_9	3.15	.732	-.014	-.174	0.6	2.1
PPETAS	PPETAS_10	3.15	.712	-.043	-.091	0.6	1.7
PPETAS	PPETAS_11	3.15	.729	.017	-.041	0.7	2.3
PPETAS	PPETAS_12	3.15	.721	-.022	-.064	0.7	2.0
EIS	EI_1	3.35	.724	-.073	-.153	0.3	3.8
EIS	EI_2	3.37	.725	.015	-.215	0.1	4.6
EIS	EI_3	3.33	.728	-.043	-.116	0.3	3.8
EIS	EI_4	3.36	.721	-.037	-.293	0.1	4.0
EIS	EI_5	3.34	.729	.054	-.019	0.3	4.7
EIS	EI_6	3.36	.728	.022	-.176	0.2	4.6
EIS	EI_7	3.35	.730	-.091	-.097	0.4	3.9
EIS	EI_8	3.34	.727	.039	-.261	0.1	4.3
EIS	EI_9	3.36	.724	.033	-.153	0.2	4.6

Note. *N = 1,824 for every item. Floor (%) is the percentage selecting the minimum response category (1), and ceiling (%) is the percentage selecting the maximum response category (5). No pronounced floor or ceiling effects were observed. Because seven-point versions were not administered concurrently, direct variance comparisons with the original response formats are not possible. Across all 29 items, the AMOS multivariate kurtosis estimate was -4.584, with a critical ratio of -2.308. PPETAS = perceived physical education teacher autonomy support; EIS = Exercise Identity Scale.*

Supplementary Table S2. Fit indices for the individual-scale confirmatory factor analyses and measurement-model comparisons

Model	χ^2	df	p	χ^2/df	CFI	TLI	RMSEA [90% CI]	RMR	SRMR
PPETAS one-factor	161.552	54	< .001	2.992	.990	.988	.033 [.027, .039]	.009	.017
PAE one-factor	3.577	2	.167	1.788	.999	.998	.021 [.000, .055]	.004	.007
EIS one-factor	46.754	27	.011	1.732	.998	.997	.020 [.010, .029]	.006	.012

Model	χ^2	df	p	χ^2/df	CFI	TLI	RMSEA [90% CI]	RMR	SRMR
EIS correlated two-factor	46.589	26	.008	1.792	.997	.996	.021 [.011, .030]	.006	.012
IPA one-factor	5.328	2	.070	2.664	.999	.996	.030 [.000, .063]	.004	.008
Correlated four-factor measurement model	477.151	371	< .001	1.286	.996	.995	.013 [.009, .016]	.008	.016
29-item single-factor model	8,529.629	377	< .001	22.625	.674	.648	.109 [.107, .111]	.055	.104

Note. *No correlated item residuals were specified, and modification indices were not used to respecify these models. For the EIS comparison, the one-factor model had AIC = 82.754 and BIC = 181.912, whereas the correlated two-factor model had AIC = 84.589 and BIC = 189.256; the two latent factors correlated at .997. CFI = comparative fit index; TLI = Tucker-Lewis index; RMSEA = root mean square error of approximation; RMR = root mean square residual; SRMR = standardized root mean square residual; AIC = Akaike information criterion; BIC = Bayesian information criterion; PPETAS = perceived physical education teacher autonomy support; PAE = physical activity enjoyment; EIS = Exercise Identity Scale; IPA = intention to be physically active.*

Supplementary Table S3. Standardized factor loadings, internal consistency, composite reliability, average variance extracted, and discriminant validity

Panel A. Standardized factor loadings and convergent validity

Construct	Item	Standardized loading	Cronbach's α	CR	AVE
PPETAS	PPETAS_1	.718	.927	.927	.515
	PPETAS_2	.720			
	PPETAS_3	.721			
	PPETAS_4	.728			
	PPETAS_5	.708			
	PPETAS_6	.733			
	PPETAS_7	.720			
	PPETAS_8	.701			
	PPETAS_9	.715			
	PPETAS_10	.728			
	PPETAS_11	.712			
	PPETAS_12	.706			
PAE	PAE_1	.685	.800	.800	.500
	PAE_2	.680			
	PAE_3	.734			
	PAE_4	.729			
EI	EI_1	.723	.910	.910	.528
	EI_2	.732			
	EI_3	.726			
	EI_4	.726			
	EI_5	.729			
	EI_6	.738			
	EI_7	.733			
	EI_8	.719			

Construct	Item	Standardized loading	Cronbach's α	CR	AVE
IPA	EI_9	.716	.814	.815	.524
	IPA_1	.702			
	IPA_2	.681			
	IPA_3	.750			
	IPA_4	.760			

Panel B. Fornell-Larcker discriminant validity matrix

Construct	PPETAS	PAE	EI	IPA
PPETAS	.718			
PAE	.405	.707		
EI	.533	.472	.727	
IPA	.488	.423	.460	.724

Note. *CR = composite reliability; AVE = average variance extracted; CFA = confirmatory factor analysis. In Panel B, diagonal entries are the square roots of AVE, and off-diagonal entries are latent-factor correlations obtained from the correlated four-factor CFA model. Every diagonal value exceeds the corresponding inter-construct correlations. PPETAS = perceived physical education teacher autonomy support; PAE = physical activity enjoyment; EI = exercise identity; IPA = intention to be physically active.*

Supplementary Table S4. Latent-variable structural equation modeling results for the hypothesized and reversed mediator orderings

Panel A. Hypothesized ordering: PPETAS → PAE → EI → IPA - structural paths

Path	B	SE	C.R.	β	p
PPETAS → PAE	.383	.027	13.955	.405	< .001
PPETAS → EI	.405	.027	14.927	.409	< .001
PAE → EI	.322	.029	10.980	.307	< .001
PPETAS → IPA	.281	.029	9.598	.295	< .001
PAE → IPA	.209	.031	6.641	.207	< .001
EI → IPA	.196	.030	6.451	.204	< .001

Panel B. Hypothesized ordering - direct and indirect associations

Association/path	Standardized estimate	Bootstrap SE	95% percentile CI	p
Direct association: PPETAS → IPA	.295	.029	[.239, .350]	< .001
PPETAS → PAE → IPA	.084	.013	[.058, .111]	< .001
PPETAS → EI → IPA	.084	.014	[.058, .111]	< .001
PPETAS → PAE → EI → IPA	.025	.005	[.017, .035]	< .001
Total indirect association	.193	—	—	—
Total association	.488	—	—	—
